# Traditional medicinal plants used for respiratory disorders in Pakistan: a review of the ethno-medicinal and pharmacological evidence

**DOI:** 10.1186/s13020-018-0204-y

**Published:** 2018-09-18

**Authors:** Alamgeer, Waqas Younis, Hira Asif, Amber Sharif, Humayun Riaz, Ishfaq Ali Bukhari, Asaad Mohamed Assiri

**Affiliations:** 1 Laboratory of Cardiovascular Research and Integrative Pharmacology, College of Pharmacy, University of Sargodha, Sargodha, Pakistan; 2 Rashid Latif College of Pharmacy, Lahore, Pakistan; 3 Department of Pharmacology, College of Medicine, King Saud University, Riyadh, Saudi Arabia; 4 Prince Abdullah Ben Khaled Celiac Disease Research Chair, Department of Pediatrics, Faculty of Medicine, King Saud University, Riyadh, Saudi Arabia

**Keywords:** Respiratory disorders, Medicinal flora of Pakistan, Pharmacological evaluation

## Abstract

Respiratory disorders are a common cause of malady and demise in Pakistan due to its remoteness, cold and harsh climatic conditions as well as scarce health care facilities. The people rely upon the indigenous plant resources to cure various respiratory disorders. The primary objective of this review was to assemble all available ethno-medicinal data of plants used for respiratory disorders in Pakistan. Pharmacological activity of these plants (based upon published scientific research), distribution, diversity, use, preparation methods, economical value, conservation status and various available herbal products of some plants have also been explored. This study scrutinized various electronic databases for the literature on medicinal plants used in Pakistan to treat respiratory disorders. A total of 384 species belonging to 85 families used to treat respiratory disorders in Pakistan has been documented. Cough was the disorder treated by the highest number of species (214) followed by asthma (150), cold (57) and bronchitis (56). Most of the plants belongs to Asteraceae (32) and Solanaceae family (32) followed by moraceae (17), Poaceae (13), and Amaranthaceae (13) with their habit mostly of herb (219) followed by Shrub (112) and tree (69). Traditional healers in the region mostly prepare ethno medicinal recipes from leaves (24%) and roots (11%) in the form of decoction. Among the reported conservation status of 51 plant species, 5 were endangered, 1 critically endangered, 11 vulnerable, 14 rare, 16 least concern, 3 infrequent and 1 near threatened. We found only 53 plants on which pharmacological studies were conducted and 17 plants being used in herbal products available commercially for respiratory disorders. We showed the diversity and importance of medicinal plants used to treat respiratory disorders in the traditional health care system of Pakistan. As such disorders are still causing several deaths each year, it is of the utmost importance to conduct phytochemical and pharmacological studies on the most promising species. It is also crucial to increase access to traditional medicine, especially in rural areas. Threatened species need special attention for traditional herbal medicine to be exploited sustainably.

## Background

Respiratory disease is a common and significant cause of illness and death around the world. In 2012, respiratory conditions were the most frequent reasons for hospital stays among children. In Pakistan acute respiratory infections constitute 30–60% of outdoor patients in hospital including 80% upper respiratory tract infections and 20% lower respiratory tract infections. The most common problems of the respiratory system are: asthma, bronchitis, common cold, cough and whooping cough [1]. Asthma affect about 300 million people worldwide and it has been estimated that a further 100 million will be affected by 2025 [2–4]. The prevalence of asthma in Pakistan is increasing day-by-day with an annual increase of 5% of which 20–30% are children. Nearly 20 million people (12%) of Pakistani adult population are already suffering from asthma while chronic bronchitis occurring in the population over 65 years of age was 14% in females and 6% in males. An estimated seven million cases of pneumonia occur every year in Pakistan and out of these, as many as 92,000 children die before their fifth birthday due to the infection.

Respiratory disorders are common in Pakistan due to its remoteness, cold and harsh climatic conditions as well as limited health care facilities. The people depend on the indigenous plant resources to treat various respiratory disorders. Herbal remedies for the treatment of respiratory disorders are common practice in many parts of the world.

Phyto-therapeutic agents are in use since ancient times for disease control but there use is greatly increased in last decade. By the end of twentieth century, 170 herbal drugs got official recognition. According to the WHO, 80% world population satisfy their primary health related needs by the use of phyto-therapeutic agents and 11% drugs are of plant origin among the essential drugs [5]. Approximately 70–95% populations of developing countries use herbal drugs for basic health care [6]. Pakistan is a rich producer of medicinal plants with more than 6000 species due to its climatic zones including high altitudes of Hindu-Kush Himalayas and Karakorum. About 600–700 (12%) species from the above mentioned figure are extensively used for medicinal purposes and various plants are also exported to foreign countries [7, 8]. From villages of Pakistan, 60% population use medicinal plants to fulfill their basic drug related needs [9].

During the previous few decades there has been an increase in the study of remedial plants and their folk usage in various parts of Pakistan. In the recent years numbers of information are documented on the use of plants in indigenous healing system either by ethnic people or rural communities around the world and Pakistanis increasing. The knowledge of ethno pharmacology and its holistic approach supported by experience can serve as a fuel for the discovery of safe, new and affordable medicines. Drugs development on the basis of natural products had an extensive history in the US, and in 1991, almost half of the drugs with maximum sale were natural products or their derivatives. With the passage of time, the emphasis on plant research is increasing day by day and stronger evidences are collected that proved the extensive use of medicinal plants in TM. Approximately 13,000 plants are investigated in previous 5 years [10].

The present study aimed at documenting the traditional uses of medicinal plants used to treat different respiratory disorders in Pakistan and to evaluate the efficacy of plant species based on the review of literature.

Specifically we sought to answer the following questions

- What is the distribution and geographical patterns of medicinal plants used for respiratory disorders across the Pakistan?
- Which plant species are most often used for treating respiratory disorders?
- Which plant parts are most commonly used in ethno preparations?
- Which preparation modes are commonly used to prepare the medicinal recipes?
- Which respiratory conditions are most commonly treated with medicinal plant species?
- Have pharmacological studies been conducted to confirm the traditional use of the medicinal plants against respiratory disorders?
- Which plant species are used in herbal preparations for the treatment of respiratory disorders?
- What is the economical and conservation status of the plant species used for respiratory disorders?

We believe that answering these questions will help to identify the plant species that have the potential to be explored in future lab trails. We also hope to highlight eventual under investigated areas.

## Materials and methods

### Data collection

Published papers up till June 2015 were retrieved from the online bibliographical databases: PubMed, Google, Google scholar, Science direct, Springer link, IUCN redlist and drug Infosys. These databases were searched by using keywords like, traditional use of plants, medicinal uses of plants, indigenous use of plants, ethno botanical surveys and ethno-pharmacological studies of different areas of Pakistan (Provinces and districts). A total of 230 scientific papers based on ethno-botanical surveys of different areas of Pakistan were reviewed for this study (Punjab = 85, KPK = 58, Sindh = 15, Baluchistan = 8, Gilgit = 22, Kashmir = 42). Plants with the reported traditional usage against respiratory diseases were screened from the data gathered. A master list was generated enlisting all the medicinal plants used in Pakistan for the treatment of respiratory disorders (Table 1). Above-mentioned databases were also searched for pharmacological studies providing supporting evidence of medicinal uses for each species. Because of the massive number of studies been consulted only reference(s) were provided and complete information on pharmacological properties can be retrieved from the original studies. All the data has been summarized in six tables and six figures.

**Table 1:** Medicinal plants use for respiratory disorders

*Scientific name*	Family	Part used	Traditional use
*Abelmoschus esculentus*	Solanaceae	Fruit	Throat, cough and bronchitis infections [43]
*Abies pindrow* Royle	Amaranthaceae	Fresh leaves	Cough, asthma and other chest infection [44]
*Abrus precatorius*	Fabaceae	Root and leaves	Asthma, cough [45]
*Acacia arabica*	Apiaceae	Leaves and fruits	Cough [46]
*Acacia jacquemontii*	Myrsinaceae	Flower, seeds, leaves, stem, bark	Asthma [47]
*Acacia modesta* Wall	Acanthaceae	Gum	Respiratory tract problems [47]
*Acacia nilotica*	Apocynaceae	Flowers	Asthma [48]
*Achillea millefolium*	Asteraceae	Leaves	Cold, flu [49]
*Achyranthes aspera* Linn.	Amaranthaceae	Leaves	Pneumonia and asthma [50]
*Aconitum chasmanthum*	Ranunculaceae	Root	Cough, and asthma [51]
*Aconitum hetrophyllum*	Ranunculaceae	Root	Cough, asthma [51]
*Aconitum violaceum*	Ranunculaceae	Root	Asthma, cough [51]
*Adhatoda vasica nees*	Capparidaceae	Whole plant	Cough, bronchitis, asthma [52]
*Adhatoda zelyanica* Medic	Apocynaceae	Whole plant	Cough, asthma [53]
*Adiantum capillus veneris*	Liliaceae	Whole plant	Coughs, bronchitis [54]
*Adiantum incisum* Forssk	Liliaceae	Fronds	Cough and cold [55]
*Adiantum venustum*	Liliaceae	Rhizome and whole plant	Cough [40]
*Aesculus indica*	Liliaceae	Fruits	Cough [40]
*Ageratum conyzoides* L.	Asteraceae	Leaves	Cold and coughs [51]
*Albizia lebbeck*	Moraceae	Bark	Flu, cough, lung problems [49]
*Alhagi maurorum* Medic	Fabaceae	Whole plant	Respiratory diseases [54]
*Allium cepa*	Apiaceae	Stem, leaves	Cough [56]
*Allium humile* Kunth	Alliaceae	Bulb infusion	Asthma/breathing, problem, cough, cold [40]
*Allium sativum* L.	Euphorbiaceae	Bulb	Respiratory tract infection [40]
*Aloe barbadensis*	Liliaceae	Whole plant	Cough, asthma [52]
*Aloe vera*	Solanaceae	Arieal parts	Cough Suppressant [55]
*Alstonia scholaris*	Fabaceae	Bark, Leaves	Asthma [57]
*Althaea officinalis* L.	Lythraceae	Flowers, leaves, roots, fruits and seeds	Asthma and bronchitis [56]
*Amaranthus viridis* L.	Caeselpiniaceae	Leaves, stem	Cough [58]
*Amaranthus albus*	Amaranthacea	Flower, stem, leaves, seeds	Asthma [56]
*Amaranthus caudatus*	Asteraceae	Shoots	Cough and asthma [59]
*Amaranthus spinosus* L.	Astraceae	Whole plant	Bronchitis [56]
*Ammi visnaga* [L.] Lam	Amaranthaceae	Fruit and flower	Bronchial asthma/breathing problems, whooping coug [56]
*Anagalis arvensis*	Poaceae	Fruit, seeds, leaves, stem, flower	Flu [60]
*Anaphalis nepalensis*	Asteraceae	Flower and leaves	Asthma, cough [61]
*Andrachne aspera*	Leguminosae	Herb	Cough, bronchitis [62]
*Anethum graveolens* L.	Solanaceae	Seeds	Bronchitis [63]
*Angelica glauaca Edgew*	Asteraceae	Ariel parts	Asthma, cold [64]
*Angelica glauca*	Umbelliferae/apiaceae	Roots	Cough [64]
*Apium Graveolens* L.	Pinaceae	Seed and root	Bronchitis, asthma [65]
*Aremisia scoparia*	Asteraceae	Whole plant	Fever cough [66]
*Arisaema flavum* Forssk.	Berberidaceae	Rhizome, fruit	Cough, cold [66]
*Arisaema jacquemonti*	Asclepiadaceae	Stem, flower	Asthma [67]
*Aristida adcensionis* L.	Poaceae	Stem, leaves	Cold [68]
*Arnebia benthamii*	Boraginaceae	Stem and leaves	Asthma, cough [69]
*Artemisia maritima* L.	Asteraceae	Aerial parts	Cough [70]
*Artemisia fragrans* Willd.	Boraginaceae	Leaves	Asthma [71]
*Artemisia macrocephala*	Euphorbiaceae		Cold, cough, flu, asthma [70]
*Artemisia scoparia*	Asteraceae	Whole plant	Cough chest problems [70]
*Artemisia vulgaris*	Asclepiadaceae	Leaves	Asthma [71]
*Asphodelus tenuifolius*	Apocynaceae	Stem, leaves, seeds	Cold [67]
*Astragalus psilocentros*	Leguminosae	Roots and thorny branches	Flue [42]
*Astragulus tragacantha*	Asteraceae	Leaves	Respiratory infection [72]
*Atropa acuminate Royle*	Ranunculaceae	Leaves, flower	Cold, flu [73]
*Avena sativa*	Poaceae	Fruit, seeds, leaves, stem	Asthma [74]
*Ayapana triplinervis*	Caryophyllaceae	Leaves	Cough [75]
*Azadirachta indica*	Solanaceae	Leaves	Cough [70]
*Bambusa bambos* [L.]	Solanaceae	Leaves herb	Expectorant [76]
*Barleria cristata* L.	Lamiaceae	Whole plant	Cold and flu [77]
*Bauhinia variegata*	Mimosaceae	Bark, root, buds	Asthma [78]
*Berberis balochistanica*	Berberidaceae	Wholeplant	Cough [79]
*Berberis lyceum*	Adiantaceae	Root, bark	Cough [78]
*Bergenia stracheyi*	Saxifragaceae	Leaves and root	Cough, asthma, lungh cancer, respiratory problem [80]
*Bergenia ciliate*	Apiaceae	Root, flowers and leaves	Coughs and colds, asthma [81]
*Bistorta amplexicaulis*	Polygonaceae	Leaves	Flu [70]
*Bistorta vivipara*	Polygonaceae	Root and stem	Chronic bronchitis [68]
*Boerhavia procumbens*	Rutaceae	Roots	Cough, asthma [70]
*Boerhavia procumbens*	Nyctaginaceae	Roots	Flue [70]
*Brassica campestris*	Chenopodiaceae	Flower, fruit, seeds, leaves, stem, pod	Cold [82]
*Broussonetia papyrifer*	Moraceae	Fruit	Cough [70]
*Bambusa arundinacea*	Solanaceae	Leaves	Cold, flu [83]
*Bunium persicum*	Apiaceae	Seeds	Cold, cough [84]
*Cadaba farinose*	Umbelliferae	Roots, leaves	Cold and cough [85]
*Calotropis gigantean*	Convolvulaceae	All parts	Cough and asthma [86]
*Calotropis procera*	Asclepiadaceae	Roots, flowers, latex	Cough [70]
*Cannabis sativa*	Cannabiaceae	Whole plant	Cough [70]
*Capparis aphylla* Roth	Euphorbiaceae	Bark	Cough and asthma [87]
*Capparis decidua*	Solanaceae	All parts	Asthma, cough [70]
*Capparis spinosa* L.	Capparidaceae	Flower and seeds	Asthma, cough [88]
*Capsella bursapastoris* L.	Brassicaceae	Seeds	Cough, respiratory diseases [88]
*Capsicum annum* L.	Asteraceae	Fruit	Bronchitis [70]
*Caragana brevifolia*	Papillionacea	Roots	Cough [80]
*Cardia myxa*	Chenopodiaceae	Flower, leaves, seed, bark	Respiratory tract infection [89]
*Carissa opaca* Stapf	Myrtaceae	Leaves, root and fruit	Cold and flu [70]
*Carthamus tinctorius* L.	Asteraceae	Flower	Cough, respiratory problems [70]
*Carum bulbocastanum* Koch.	Apiaceae	Seeds	Fiue [90]
*Carum capticum* L.	Violaceae	Seeds, leaves and flowers	Cough bronchitis and diarrhea [91]
*Carum carvi* L.	Asteraceae	Stem and leaves	Bronchitis, cough [92]
*Carum copticum* Benth	Solanaceae	Whole plant	Whooping cough [22]
*Cassia Occidentalis*	Mimosaceae	Leaves	Cough [22]
*Cassia fistula* L.	Moraceae	Fruit	Cough and flue [93]
*Catharanthus roseus*	Mimosaceae	Whole plant	Cold, flue, bronchitis [70]
*Celtis australis* L.	Ulmaceae	Leaves	Cough [94]
*Chenopodium album*	Fabaceae	Flower, fruit, seeds, leaves, stem	Cold [74]
*Chenopodium botrys* L.	Chenopodiaceae	Stem and leaves	Asthma [67]
*Chenopodium morale*	Fabaceae	Flower, fruit, seeds, leaves, stem	Flu [62]
*Cicer arietinum* L.	Moraceae	Fruit	Flu, cough [62]
*Cichorium intybus* L.	Asteraceae	Whole plants	Asthma and breathing problems [70]
*Cichorium endivia* Linn.	Asteraceae	Seeds	Cough [94]
*Cistanche tubulosa*	Poaceae	Whole plant	Cough [95]
*Citrullus colocynthis*	Cucurbitaceae	Leaves, fruits	Bronchial asthma [93]
*Citrus medica*	Solanaceae	Leaves, seeds and latex	Cough, cold, asthma [70]
*Colchicum luteum*	Colchicaceae	Coms	Bronchial diseases [86]
*Convolvulus arvensis* Linn.	Astraceae	Whole plant	Cough, flu [67]
*Conyza bonariensis*	Brassicaceae	Whole plants, oil	Bronchial complaints [96]
*Conyza canadenisis*	Chenopodiaceae	Whole plant	Bronchial catarrh [52]
*Cordia dichotoma*	Moraceae	Whole plant and fruit	Dry cough [97]
*Cordia gharaf* Ehrenb.	Oleaceae	Tree fruit	Dry cough [98]
*Cordia obliqua* Willd.	Anacaediaceae	Fruits	Throat infection, common cold [93]
*Cordial dichotoma*	Boraginaceae	Leaves	Asthma [99]
*Coriandrum sativum*	Brassicaceae	Flower, fruit, seeds, leaves, stem	Respiratory tract infection [70]
*Coronopus didymus*	Brassicaceae	Leaves and tender parts	Asthma, bronchitis [67]
*Corydalis ramose*	Fumariaceae	Leaves	Cough [100]
*Cousinia stocksii* C. Winkler	Asteraceae	Gum and roots	Asthma [101]
*Cucurbita maxima*	Polygonaceae	Seeds	Cough [38]
*Cupressus sempervirens*	Asteraceae	Fruit and seed	Flu and cold [70]
*Cuscuta reflexa*	Fabaceae	Whole plant	Cough [70]
*Cydonia oblonga* Mill	Violaceae	Fruit	Cough [102]
*Cymbopogon jawaracusa*	Salvadoracea	Whole plant	Respiratory diseases [103]
*Cymbopogon jwarancusa*	Poaceae	Leaves, flowers and roots	Flu, and cough [82]
*Cynodon dactylon*	Ranunculaceae	Leaves	Asthma [94]
*Cynoglossum lanceolatum*	Solanaceae	Whole plant	Bronchitis, Cough [67]
*Datura stramonium*	Solanaceae	Seeds, flowers, leaf, fruit	Whooping cough [93]
*Datura alba*	Zygophyllaceae	Leaves and seeds	Asthma [103]
*Datura fastuosa* L.	Solanaceae	Whole plant	Asthma [70]
*Datura innoxia* Mill	Euphorbiaceae	Dried leaves, seeds and fruit	Asthma [70]
*Datura metel* Linn.	Solanaceae	Whole plant	Asthma [70]
*Daucus carota*	Moraceae	Stem, root, carrot	Asthma, bronchitis [99]
*Delphinium brunonianum* Royle	Ranunculaceae	Leaves, flower	Cough, asthma [88]
*Dendrocalamus strictus*	Scrophulariaceae	Leaves	Cough and cold [82]
*Desmodium gangeticum*	Caesalpiniaceae	Roots	Asthma and cough [45]
*Desmostachya bipinnata*	Nyctaginaceae	Leaves, root	Asthma [70]
*Diospyros lotus* L.	Punicaceae	Flower	Cough [104] ]
*Dipterygium glaucum*	Euphobiaceae	Areal part	Asthma [93]
*Dodonaea viscosa*	Spaindaceae	Leaves, flowers and seeds	Chest infection [74]
*Duchesnea indica*	Rosaceae	Aerial parts, fruits	Cough [80]
*Echinops echinatus*	Capparidaceae	Roots	Cough [78]
*Eclipta prostata* Linn.	Boraginaceae	Whole plant	Flu [70]
*Elaeagnus angustifolia* L.	Elaeagnaceae	Fruits	Respiratory problems [70]
*Elaeagnus parvifolia*	Fabaceae	Shrub	Cough [105]
*Emblica officinale* Gaerth	Euphorbiaceae	Tree	Cold, cough [44]
*Ephedra gerardiana*	Ephedraceae	Stem	Respiratory disorders, asthma/breathing problem [84]
*Ephedra intermedia*	Ephedraceae	Whole plant	Asthma and tuberculosis [70]
*Ephedra procera*			Cough and asthma [70]
*Ephedra ciliata*	Ephedraceae	Wholeplant	Chest problems, cough, asthma [84]
*Eucalyptus citirodora*	Solanaceae	Leaves	Cold, flue, and cough [93]
*Eucalyptus globulus* Labill	Moraceae	Tree	Flue [70]
*Eugenia jambolana*	Solanaceae	Bark	Bronchitis, asthma [87]
*Euphorbia helioscopia* Linn.	Fabaceae	Whole plant	Asthma, bronchitis, cough [60]
*Euphorbia tircucalli*	Mimosaceae	Juice	Cough, asthma [60]
*Euphorbia hirta*	Labiatae	Whole plant	Asthma, chronic bronchial [93]
*Euphorbia prostate*	Fabaceae	Whole plan	Asthma [87]
*Euphorbia thymifolia*	Salvadoraceae	Whole plant	Bronchial affection, cough and asthma [70]
*Evolvulus alsinoides*	Euphorbiaceae	Whole plant	Bronchitis [87]
*Fagonia bruguieri* DC	Zygophyllaceae	Whole plant	Asthma [106]
*Fagonia cretica* L.	Solanaceae	Whole plant	Antiasthematic, cough [94]
*Fagonia indica* Burm. F	Amaranthaceae	Whole plant	Asthma [70]
*Ferula assa*-*foetida*	Apiaceae	Root, stem and gum resin	Cough, asthma [70]
*Ferula narthex* Boiss.	Malvaceae	Whole plant	Cough and asthma [70]
*Ferula oopoda* [Boiss. and Buhse]	Apiaceae	Seeds, leaves and sap	Cough [107]
*Ficus benghalensis*	Moraceae	Milk of leaves, bark, root	Asthma [70]
*Ficus religiosa* L.	Papilionaceae	Fruit, leaves	Asthma [78]
*Ficus carica* L.	Moraceae	Fruit and leaves	Cough [93]
*Ficus elastic*	Moraceae	Bark, fruits and leaves	Cough, asthma [93]
*Ficus lyrata*	Molluginaceae	Whole plant	Asthma, cough [70]
*Ficus palmate*	Moraceae	Fruit, latex	Asthma, cough [70]
*Foenicullum vulgare* Miller	Papilionaceae	Seed and leaves	Cough, pneumonia [108]
*Foeniculum capillacerm*	Asteraceae	Seed, root, leaves	Cough, and asthma [45]
*Fragaria nubicola*	Rosaceae	Root and fruit	Asthma [81]
*Fritillaria roylei* Hook.	Asteraceae	Herb	Broncho-asthma [103]
*Fumaria indica*	Fumariaceae	Whole plant	Cough [109]
*Gentiana kurrooroyle*	Gentianaceae	Flower	Cough [110]
*Gentianodes olivieri*	Gentianaceae	Whole plant	Cough, chest problems [81]
*Gentianodes tianschanica*	Gentianaceae	Leaves	Cough [81]
*Glossonema varians*		Fruit	Cough [111]
*Glycyrrhiza glabra*	Adiantaceae	Roots	Cough [70]
*Grewia optiva*	Ranunculacea	Leaves	Cough [59]
*Hackelia uncinatum*	Ranunculaceae	Flowers	Coughs [103]
*Helianthus annuus*	Papilionaceae	Flower, root, seed, leaves	Asthma, bronchial [81]
*Helianthus tuberosus*	Acanthaceae	Tubers	Cough and bronchitis and flu, respiratory diseases [83]
*Heliotropium europaeum*	Malvaceae	Whole plant	Cough [86]
*Heracleum candicans*	Apiaceae	Root	Asthma, cough [112]
*Hippophae rhamnoides*	Elaeagnaceae	Fruit juice	Cough [70]
*Hyoscyamus niger* Linn.	Zygophyllaceae	Whole plant	Asthma, whooping cough [70]
*Hyoscyamus insanus* Stocks	Caesalpiniaceae	Whole plant	Anti asthmatic [45]
*Inula grantioides*	Asteraceae	Whole plant	Asthma [107]
*Inula racemosa* Hook	Violaceae	Root	Asthma and bronchitis [100]
*Ipomea carnea*	Malvaceae	Leaves, stem	Asthma [113]
*Iris hookeriana*	Iriddaceae	Flower	Asthma, cough and bronchitis [68]
*Jatropha curcas* L.	Malvaceae		Bronchitis [94]
*Juglans regia*	Euphorbiaceae	Fruits	Asthma [70]
*Juniperus excelsa* M. B.	Cupressaceae	Seeds and leaves	Chest infection [84]
*Justicia adhatoda* L.	Alliaceae	Cold	Cough, cold, flu [70]
*Lactuca serriola* L.	Asteraceae	Whole plant	Whooping cough and asthma [70]
*Laepus nigricollis*	Bouidugs		Bronchial diseases [62]
*Lantana camara*	Amaranthaceae	Leaves, root and flowers	Respiratory diseases [48]
*Lasiurus scindicus*	Poaceae	Stem, leaves	Cough [74]
*Lathyrus aphaca* L.	Moraceae	Shoot	Hiccough [95]
*Launea procumbus*	Euphorbiaceae	Whole plant	Cold, flu, cough [57]
*Lawsomia alba* Lam	Meliaceae	Leaves	Bronchitis [114]
*Lawsonia inermis alba*	Punicaceae	Powdered leaves, seeds, bark and flowers	Cough, bronchitis [57]
*Lemna minor*	Convolvulaceae	Whole plant	Cough [115]
*Lepidium sativum* L.	Fabaceae	Shoot	Cough and cold [95]
*Leptadenia pyrotechnica*	Brassicaceae	Root, bark and leaves	Asthma [76]
*Limeum indicum*	Nyctaginaceae	Leaf and stem	Cold [95]
*Linum usitatissimum*	Papaveraceae	Seed, bark, leaves, flower and Oil	Cough, asthma [52]
*Lonicera periclymenum* L.	Caprifoliaceae	Leaves and flower	Cough [116]
*Lychnis coronaria* Lamak	Anacaediaceae	Roots and flowers	Lung troubles [117]
*Malva neglecta*	Solanaceae	Leaves and stem	Bronchitis, cough [8]
*Malva parviflora*	Cruciferae	Whole plant	Cough [118]
*Malva sylvestris*	Myrtaceae	Whole plant	Chronic bronchitis [84]
*Malvastrum coromendelianum*	Oxalidaceae	Flowers	Coughs [109]
*Mangifera indica*	Capparaceae	Flowers, leaves, kernel, bark, fruits	Asthma, cough [93]
*Marrubium vulgare* L.	Umbelliferae	Leaves	Cough [51]
*Medicago denticulate*	Poaceae	Seeds	Respiratory diseases [119]
*Melia azodirachta* L.	Salvadoraceae	Root	Lung complaints [120]
*Melilotus indica* L.	Verbenaceae	Annual herb	Bronchial disorder [109]
*Melilotus parviflora*	Lamiaceae	Whole plant, seeds	Cold [53]
*Mentha longifolia*	Labiateae	Leaves	Cough [52]
*Mentha royleana* Benth.	Lamiaceae	Leaves	Cough and cold [57]
*Micromeria biflora*	Fabaceae		Colds and coughs [121]
*Mimosa pudica* L.	Lamiaceae	Roots and leaves	Asthma [122]
*Mollugo cerviana*	Oxalidaceae	Fruit, stem, leaves	Asthma [75]
*Momordica balsamica* L.	Moraceae	Fruits	Asthma [103]
*Momordica charantia* L.	Poaceae	Fruit	Treat cough, bronchitis [123]
*Morus alba* L.	Solanaceae	Flower, leaves, root, bulb	Cough [120]
*Morus nigra* L.	Poaceae	Leaves, roots, fruits	Cough [46]
*Mukia moderaspatana*	Fabaceae	Flower, seeds, stem, leaves	Cough [118]
*Murraya koenigii*	Rosaceae	Leaves	Asthma [122]
*Musa paradisica* L.	Amaranthaceae/Chenopodiaceae	Leaves, flower	Whooping cough [103]
*Nasturtium officinale* R.Br.	Rosaceae	Leaves	Chest troubles [124]
*Nepeta praetervisa* Rech. F.	Lamiaceae	Leaves	Cold, chest problems [84]
*Nigella sativa*	Ranunculacea	Seeds	Whooping cough [43]
*Nonea edgeworthii*	Cucurbitaceae	Leaves	Cough [103]
*Nyctanthes arbor*-*tristis* L.	Asclepidiaceae	Shrub flowers	Cough [73]
*Ocimum basilicum*	Solanaceae	Leaves and seeds	Bronchitis, cough, cold [52]
*Oenothera rosea* L.	Lamiaceae		Whooping cough [70]
*Olea ferruginea*		Leaves	Cough, cold, flue [103]
*Onosma hispida*	Boraginaceae	Leaves, flower	Cough, respiratory diseases [81]
*Onosma bracteatum* Wall	Moraceae	Whole plant	Asthma and bronchitis [125]
*Opuntia dillenii*	Lythraceae	Fruits	Asthma, whooping cough [83]
*Opuntia monacantha* Haw	Caesalpinaceae	Whole plant	Bronchitis and asthma [126]
*Origanum vulgare*	Violaceae	Perennial herb	Respiratory problems, colds, flu, asthma [53]
*Oryza sativa*	Salicaceae	Fruit, leaves, stem	Cold [103]
*Oxalis corniculata* L.	Ranunculaceae	Leaves	Respiratory disorders like bronchitis, asthma [103]
*Oxystelma esculentum*	Asclepiadaceae	Fruits	Expectorant, cough [93]
*Panicum antidotale* Retz	Polygonaceae	Stem, leaves	Cough [103]
*Papaver hybridum*	Solanaceae	Petals	Flu and cough [86]
*Papaver nudicaule* L.	Papaveraceae	Herb	Cough [120]
*Papaver somniferum* L.	Papaveraceae	Seed, fruit	Cough [107]
*Peganum harmala*	Solanaceae	Seeds, leaves	Asthma [52]
*Pennisetum typhoides* Burm.	Poaceae	Seeds	Flu and cough [78]
*Periploca aphylla*	Rhamnaceae	Whole plant	Nasal decongestant [54]
*Phalaris minor* Retz	Solanaceae	Leaves, stem	Cold, cough [103]
*Phoenix dactylifera*	Capparaceae	Fruit, gum and seeds	Colds, bronchial catarrh [109]
*Phyla nodiflora*	Verbenaceae	Whole plant	Cold [93]
*Phyllanthus emblica*	Liliaceae	Fresh, fruits, seeds, flowers, leaves, bark	Asthma, bronchitis [70]
*Picrorhiza kurroa*	Scrophulariaceae	Root	Asthma [68]
*Pimpinella diversifolia*	Asteraceae	Fruit	Cough, cold [103]
*Pinus roxburghii*	Solanaceae	Areal part	Coughs, cold [44]
*Pistacia atlantica*	Anacardiaceae	Gum	Cough, Chestproblems [70]
*Pistacia integerrima*	Rosaceae	Leaf galls	Cough, asthma [70]
*Pistacia khinjuk*	Anacardiaceae	Fruits	Cough [84]
*Plantago lanceolata* L.	Plantaginaceae	Leaves and seeds	Cough and chest diseases [70]
*Plantago major* L.	Punicaceae	Leaves, stem	Asthma, cough [70]
*Plantago ovata* Forssk	Brassicaceae	Seeds and husk	Cough and cold [101]
*Plantago lanceolata*	Plantaginaceae	Fresh or dried leaves	Relieving coughs [70]
*Polygonum affine*	Polygonaceae	Root	Lung disorder [110]
*Polygonum hydropiper* L.	Polygonaceae	Aerial parts	Respiratory [90]
*Populus tremula*	Xanthorhoeaceae	Leaves, bark	Cough [74]
*Portulaca oleracea*	Asteraceae	Aerial part of plant	Asthma [86]
*Portulaca quadrifida*	Tamaricaceae	Leaves	Cold, flu, Respiratory problems [70]
*Potentilla bifurca* L.	Rosaceae	Aerial part	Cough [57]
*Potentilla salesoviana*	Rosaceae	Flower	Cough, cold [57]
*Primula veris* L.	Primulaceae	Flower	Bronchitis [127]
*Prosopis cineraria*	Moraceae	Fruit, pods	Asthma [70]
*Prosopis juliflora*	Asclepidaceae	Xerophytic shrub	Asthma, cough [70]
*Prosopis spicigera*	Cactaceae	Bark, leaves, flowers	Asthma [109]
*Prunella vulgaris* L.	Asteraceae		Difficult breathing [70]
*Prunus cornuta* L.	Elaeagnaceae	Fruit	Asthma [92]
*Psammogeton biternatum*			Cough [101]
*Pseudognaphalium luteoalbum*	Asteraceae	Leaves	Asthma/breathing problem [110]
*Psidium guajava*	Malvaceae	Fruit	Old cough, bronchitis and chronic whooping cough [70]
*Punica granatum*	Punicaceae	Roots, fruit, rinds	Cough [70]
*Pyrus communis* L.	Rosaceae	Fruits	Cough [89]
*Quercus incana Bartram*	Podophyllaceae	Bark and fruits	Asthma/breathing problems [89]
*Quercus leucotrichophora*	Plantaginaceae	Banafsha	Asthma, cough [128]
*Quercus floribunda*	Rhamnaceae	Seeds	Asthma [70]
*Ranunculus arvensis* L.	Acanthaceae	Whole plant	Asthma [22]
*Ranunculus muricatus* L.	Solanaceae		Asthma [103]
*Raphanus sativus* L.	Brassicaceae	Whole plant	Asthma [81]
*Rheum australe* D. Don	Polygonaceae	Roots, rhizomes, stem, leaves	Cough [70]
*Rhazya stricta*	Asclepiadaceae	Whole plant	Asthma [70, 129]
*Rheum emodi*	Rosaceae	Floral scape	Cough and flu [38]
*Rheum spiciforma* Royle	Polygonaceae	Roots	Chronic bronchitis, asthama [70]
*Rhodiola imbricate* Edgew	Crassulaceae	Root	Cough [110]
*Rhus coriaria* L.	Apocynaceae	Leaves, flower, root	Cough, asthma [130]
*Rhynchosia minima*	Mimosaceae	Leaves	Asthma [93]
*Ricinus communis*	Leporidae	Leaves	Asthma and cough [49]
*Rosa damascene*	Amaranthaceae		Bronchitis, cough [76]
*Rosa indica* L.	Solanaceae	Flowers	Asthma [131]
*Rosa webbiana*	Umbelliferae	Fruits	Asthma [51]
*Rubus fruiticosus* Hook.	Violaceae		Whooping cough [128]
*Rubus ulmifolius*	Rosaceae	Leaves	Cough [59]
*Rumex crispus* L.	Solanaceae		Cough [103]
*Rumex dentatus* L.	Tamaricaceae	Fruit, stem, leaves	Cold [70]
*Rumex hastatus* D. Don	Fagaceae	Whole plant	Asthma, cough [130]
*Rumex nepalensis* Spreng	Polygonaceae	Leaves, roots	Lungs diseases [103]
*Saccharum bengalense*	Violaceae	Stem	Cough [56]
*Salsola baryosma*	Amarylliadaceae	Stem, leaves	Cough [74]
*Salvadora oleoidesdecne*	Apiaceae	Stem, root, oil, seed, leaves, bark	Cough [46]
*Salvadora persica* L.	Salvadoraceae	Seeds, roots	Cough [107]
*Salvia nubicola*	Labiateae	Leaves	Cough, asthma and other respiratory issues [81]
*Salvia moorcroftiana*	Malvaceae	Root	Cough [52]
*Salvia officinalis* Linn.	Liliaceae	Cough and asthma	Cough, cold [132]
*Saussurea atkinsonii*	Asteraceae	Aerial parts	Respiratory diseases like asthma, cough [133]
*Saussurea ceratocarpa*	Asteraceae	Whole plant	Asthma, bronchitis [68]
*Scorzonera tortuosissima*	Asteraceae	Roots, gum, flower, leaves	Cough and chest problems [107]
*Sema alexandriana Miller*	Asclepiadaceae	Dried leaves and pods	Asthma [52]
*Silybum marianum*	Sapindaceae	Leaves	Flu [35]
*Sisymbrium irio* L.	Brassicaceae	Leaves	Cough [52]
*Skimmia laureola*	Solanaceae	Leaves	Asthma [128]
*Solanum melongena*	Adiantaceae	Fruit, leaf, root	Asthma, bronchitis [56]
*Solanum surratense*	Solanaceae	Berries, root, fruit	Cough, asthma [134]
*Solanum tuberosum*	Solanaceae	Leaf, flower and tuber	Cough [134]
*Solanum nigrum* L.	Solanaceae	Leaf, berries, flowers, root	Cough, bronchitis [70]
*Solanum incanum* L.	Acanthaceae	Leaves, seeds	Bronchitis [52]
*Sonchus asper*	Leguminosae	Whole plant	Cough, asthma [70]
*Spinacia oleraceal*	Amarylliadaceae	Leaves	Cough [103]
*Sporobolus ioclados*	Solanaceae	Stem, leaves	Cough [103]
*Stacia integerrima*	Scrophulariaceae	Bark and fruit	Bronchial disorder [103]
*Stellaria media*	Caryophylaceae	Herb	Cough [103]
*Suaeda fruiticosa*	Fabaceae	Flower, fruit, stem, leaves	Cough [103]
*Sussurea lappa*	Compositae	Root	Cough with cold [94]
*Swertia cordata*	Gentianaceae	Flower	Cough [110]
*Swertia petiolata*	Violaceae		Asthma, bronchitis [70]
*Tamarix aphylla*	Amaranthaceae	Whole plant	Cough [135]
*Tamarix dioica*	Acanthaceae	Bark	Cough [136]
*Tamarix gallica*	Zygophyllacaea	Flower, fruit, stem, leaves	Asthma [58]
*Tanacetum senecionis*	Asteraceae	Floral parts	Asthma [57]
*Taverniera persica*	Myrsinaceae	Fruit, seeds, leaves, stem	Cough [74]
*Taxus baccata* Linn.	Moraceae	Bark	Asthma and bronchitis [100]
*Taxus wallichiana Zuce*	Scrophulariaceae	Leaves and fruits	Pneumonia, bronchitis, whooping cough, asthma [128]
*Tephrosia lupinifolia*	Myrtaceae	Roots, leaf, stem bark	Asthma [70]
*Thymus linearis*	Labiateae	Arial parts	Cough, asthma [128]
*Thymus serpyllum* L.	Lamiaceae	Dried leaves	Whooping cough, asth ma and respiratory inflammation [70]
*Trachyspermum ammi*	Malvaceae	Seeds and oil	Bronchitis, asthma and colds, cough [52]
*Trianthema portulacastrum* L.	Amaranthaceae	Roots	Asthma [70]
*Trianthema triquetra*	Chenopodiaceae	Flower, fruit, leaves, stem	Asthma [74]
*Tribulus longipetalus* L.	Amaranthaceae	Stem, leaves, fruit, seeds	Flu [51]
*Tribulus terrestris* L.	Amaranthacea	Root and fruit	Cough, asthma [70]
*Trichodesma africanum*	Boraginaceae	Leaves and fruits	Cough and chest problems [107]
*Trichodesma indicum*	Boraginaceae	Leaves and flowers	Flue and cough [70]
*Trifolium resupinatum*	Malvaceae	Whole plant	Whooping cough [70]
*Trifolium alexandrium*	Poaceae	Stem, leaves	Respiratory tract [74]
*Trifolium pratense* L.	Fabaceae	Dried flowers	Whooping cough, bronchitis and asthma [70]
*Trifolium repens*	Lamiaceae	Perennial herb	Coughs, colds [70]
*Trigonella foenumgraecum*	Acanthaceae		Cough [70]
*Tussilago farfara* L.	Asteraceae	Leaves	Cough, respiratory problems [70]
*Tylophora hirsuta* L.	Poaceae	Root, leaves	Asthma and whooping cough [70]
*Verbascum thapsus* L.	Scrophulariaceae	Flowers and leaves	Cough [128]
*Vernonia anthelmentica*	Asteraceae	Seeds	Cough, chest infection, Pneumonia [76]
*Vicia sativa*	Primulaceae	Whole plant	Respiratory diseases [70]
*Viola serpens*	Violaceae	Whole plant	Cold, cough and flu [70]
*Viola betonicifolia* Sm	Asclepiadaceae	Whole herb and flowers	Lung troubles, cough and colds, bronchitis [70]
*Viola biflora* L.	Pteridaceae	Flower	Cold and flu [22]
*Viola canescens *Wall. ex	Violaceae	Floral part	Cough [70]
*Viola fedtschenkoana*	Violaceae	Whole plant	Cough [22]
*Viola odorata* L.	Berberidaceae	Whole Plant	Flu and cold [22]
*Viola stacksii*	Acanthaceae	Whole plant	Cold, cough [70]
*Viola sylvatica Fries*	Acanthaceae	Dried plant	Cough, and cold [70]
*Vitex negundo*	Verbenaceae	Leaves	Flu [50]
*Vitis vinifera* L.	Rhamnaceae	Flowers	Bronchitis [133]
*Wattakaka volubilis*	Phasianidae	Leaves	Cough, cold and other respiratory problems [70]
*Withania coagulans*	Meliaceae	Fruit	Cough, asthma [74]
*Withania somnifera*	Zingerberaceae	Fruit, seeds, leaves, stem, flower	Flu [52]
*Zataria multiflora* Boiss.	Lamiaceae	Stem and leaves	Cough and chest problems [101]
*Zea mays* L.	Poaceae		Cough problems [44]
*Zaleya pentandra*	Aizoaceae	Root	Cough, phlegmatic cough and flue [70]
*Zingiber officinalis*	Asclepiadaceae	Stem, leaves	Flu [70]
*Ziziphus jujube* Mill.	Liliaceae	Fruits	Bronchitis [93]
*Zizyphus nummularia*	Solanaceae	Fruit	Bronchitis [40]
*Zizyphus sativa G*	Rhamnaceae	Fruit and leaves	Bronchitis [104]

### Data analysis

Respiratory disorders have been divided into 12 categories depending upon the diseases enlisted in published research articles on ethno botanical survey of Pakistan. Diseases or categories consisting of similar disorders or pharmacological effects have been grouped as single category. The plant list was prepared on the Pakistan level as a whole by enlisting each plant only once that is being reported in different provinces for the same respiratory disease.

The conservation status of plant species was determined following the IUCN red list categories and criteria version 3.1 (IUCN red list categories and criteria, 2001) and economical value of plant species were determined using scientific literature based on the commercial value of medicinal plants in Pakistan.

## Results and discussion

Diversity of plants remains essential for human beings, providing numerous modern and traditional remedies to the healthcare system. It can be precisely assumed that the present day ethno-botanical pharmacology is as old as man himself. Different medicinal plants have been in use since the ancient time. Even in the present age of science and technology, people in the developed countries still rely on traditional system of healthcare not only because of its low price, but also due to very less side effects, as compared to the modern allopathic medicines. Pakistan is rich in natural sources including medicnal plants and most of inhabitants are in remote areas and have limited economical sources so they rely on the plants for their health care needs.

### Ethno botanical surveys and distribution of medicinal plants

**Fig. 1:**
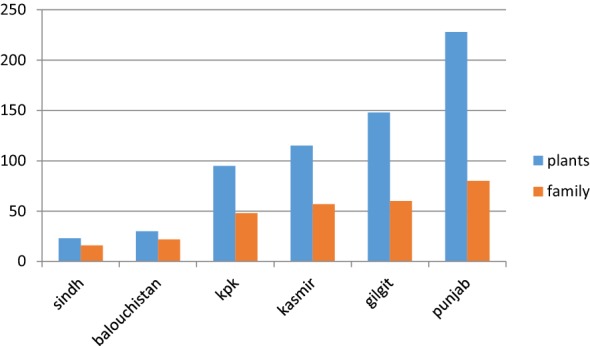
Distribution of medicinal plants in different regions of Pakistan

Pakistan has been bestowed with distinctive biodiversity, consisting of a variety of climates, topographical regions, and ecological zones and holds rich diversity of medicinal plants used against various ailments [11]. The present review reported 385 plants of 85 families from different regions of Pakistan being ethno-medicinally used for treating different types of respiratory problems. Majority of 228 plants of 80 families were reported from Punjab followed by 148 plants of 60 families from Gilgit, 115 plants of 57 families from Kashmir, 95 plants of 48 families from KPK, 30 plants of 22 families from Baluchistan and 23 plants of 16 families from Sindh. Many of the plants were used in more than one region; those plants were counted just one time while enlisting. Literature review elucidates that majority of plant species being used for respiratory disorders in Pakistan belongs to Punjab. This botanical diversity from Punjab might be owing to its varied climate and soil types [12]. The distribution of plants in different regions of Pakistan is shown in Fig. 1.

A large portion of Ethno botanical overview have been accounted for from 30 districts of Punjab followed by 18 districts of Khyber Pakhtunkhwa, 12 districts of Baluchistan, 9 districts of Sindh, 7 districts of Gilgit Batistan, 10 districts of Kashmir. Still there are number of under-investigated districts that need to be surveyed for ethno botanical studies including 6 districts of Punjab, 8 districts of Khyber Pakhtunkhwa, 20 districts of Baluchistan, 20 districts of Sindh and 1 district of Gilgit Batistan. Another reason for majorty of the plants from Punjab might be that ethnobotanical surveys have been reported from maximum areas of Punjab except a few, while some regions of Northern areas and many areas of Balochistan and Sindh have not so far been explored concerning ethnobotanical knowledge. A list of investigated and under-investigated districts has been mentioned in Table 2.

**Table 2:** Investigated and under-investigated districts for ethno-botanical studies

Province	Investigated districts for ethno-botanical studies	Under-investigated districts for ethno-botanical studies
*Punjab*	30	6
	Attock, Bahawalnaga, Bahawalpur, Bhakkar, Chakwal, Dera Ghazi Khan, Faisalabad, Gujranwala, Gujrat, Jhang, Jhelum, Kasur, Khushab, Mianwali, Multan, Muzaffargarh, Narowal, Nankana Sahib, Pakpattan, Rajanpur, Rawalpindi, Sahiwal, Sargodha, Sialkot, Toba Tek Singh, Vehari	Chiniot, Hfizabad, Khanewal, Okara, Rahim Yar Khan, Sheikhupura
*Khyber Pakhtunkhwa*	18	8
	Abbottabad, Bannu, Battagram, Buner, Chitral, Dera Ismail Khan, Haripur, Karak, Kohat, Upper Kohistan, LakkiMarwat, Lower Dir, Malakand, Mansehra, Peshawar, Swat, Upper Dir, Lower Kohstan	Charsadda, Hangu, Mardan, Nowshera, Shangla, Swabi, Tank, Tor Ghar
*Sindh*	9	20
	Ghotki, Jamshoro, Karachi, Kairpur, Sanghar, Sukkur, Tharparkar, Thatta, Karachi West	Badin, Dadu, Hyderabad, Jacobabad, Kashmore, Larkana, Matiari, Mirpurkhas, NaushahroFiroze, ShaheedBenazirabad. Kambar, Shahadkot, Shikarpur, TandoAllahyar, Tando Muhammad Khan, Umerkot, Sujawal, Karachi Central, Karachi East, Karachi South, Korangi, Malir
*Gilgit Baltistan*	7	1
	Ghanche, Skardu, Astore, Diamer, Ghizer, Gilgit, Hunzanagar	Kharmang
*Kashmir*	10	
	Muzaffarabad, Hattian, Neelum, Mirpur, Bhimber, Kotli, Poonch, Bagh, Haveli, Sudhnati	
*Baluchistan*	12	20
	Awaran, Barkhan, Kachhi (Bolan), Chagai, Gwadar, Kalat, Khuzdar, Lasbela, Mastung, Musakhel, Quetta, Ziarat	Dera Bugti, Harnai, Jafarabad, Jhal Magsi, Kech (Turbat), Kharan, Kohlu, Killa Abdullah, Killa Saifullah, Loralai, Nasirabad, Nushki, Panjgur, Pishin, Sherani, Sibi, Washuk, Zhob, Lehri, Sohbatpur

### Diversity, habit, and part used of medicinal plants

**Fig. 2:**
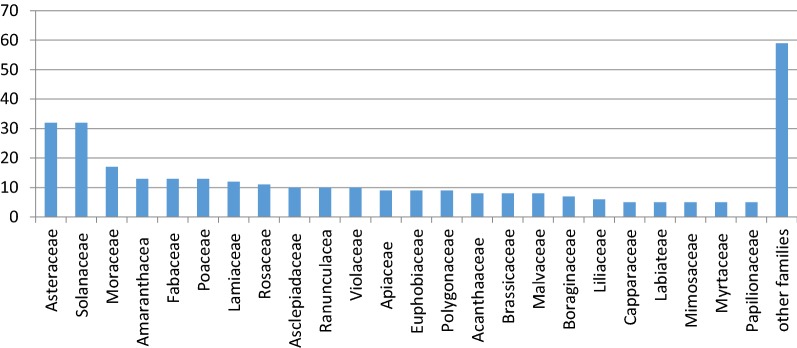
Most commonly found families

A total of 384 medicinal plants of 85 families were found in the literature that are being employed for the treatment of respiratory diseases in Pakistan. The most commonly used plants were member of *Asteraceae* family (32) followed by *solanaceae* (32), *moraceae* (17), *Poaceae* (13), *Fabaceae* (13), *Amaranthaceae* (13), *Lamiaceae* (12), *rosaceae* (11), *Violaceae* (10), *ranunculaceae* (10), *Asclepiadaceae* (10), *Euphorbiaceae* (9), *apiaceae* (9), *polygonaceae* (9), *Malvaceae* (8), *Acanthacea* (8), *brassicaceae* (8), *Boraginaceae* (7), *liliaceae* (6), *Capparaceae* (5), *Labiatae* (5), *Mimosaceae* (5), *Papilionaceae* (5), *Myrtiaceae* (5) and 10 families contain 4 plants, 9 families containg 3 plant species, 12 families consisting of 2 plant species and 28 families contain 1 plant species. The results, in terms of percentage, of plants in each family are represented in Fig. 2. *Asteraceae* holds the top position among the families used in ethno-medicines which indicates the presence of effective bioactive ingredients in the members of this family [13]. This predominance could be explained by worldwide highest number of species (23,000 species and 1535 genera) of this diverse family found in almost every habitat of all countries except Antarctica [14, 15]. Various secondary metabolites have been reported to be present in the members of this family especially sesquiterpene lactones, in addition to volatile oils and terpenoids [16, 17]. Perhaps these secondary metabolite profiles, together with the large number of species, are primarily responsible for the relevance of this family in traditional medicine. The prevalence of asteraceae family in medicinal use is not a new finding as studies from various other countries also reported similar results [18, 19]. Many species of asteraceae family are typically identified as weeds occurring in anthropogenic environments and are among the first species to emerge in the field after the soil is prepared for planting. This may contribute to the high rate of citations of species of this family in rural communities where the home gardens are the main source of medicinal plants [20].

**Fig. 3:**
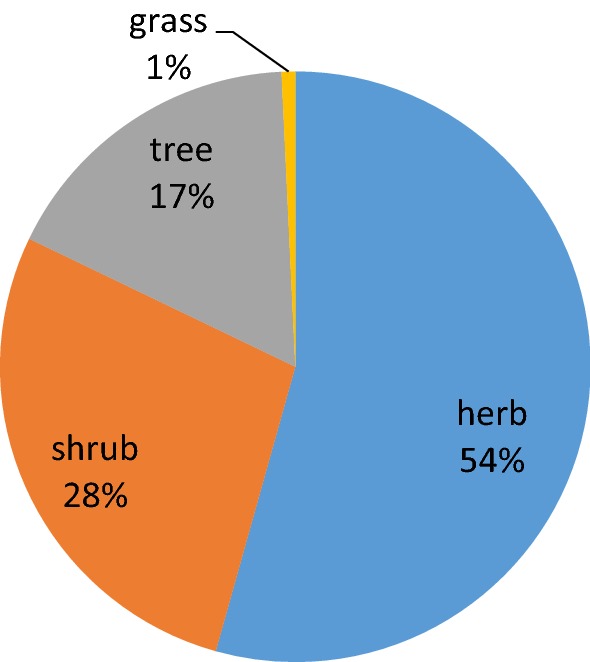
Habit of medicinal plant species

Most of the medicinal plants used in ethno medicine for treating the respiratory disorders are herbs (219) followed by Shrub (112), tree (69) and only three (3) plant species are being documented as grass. Habit of plants in different regions of Pakistan is shown in Fig. 3. This predominance of herbs as a source of herbal therapies is often attributed to the fact that their high ethno botanical studies could be an indication of their abundance easy availability and the traditional knowledge [21]. Whilst shrubs and trees seem to be preferred because of their availability round the year and they are resistant to drought and seasonal variations [22]. Majority of herbal recipes include trees and shrubs due to their easy accessibility round the year, followed by utilization of herbs which might be related to their easy collection methods, higher abundance and efficacy in curing ailments as compared to other life forms [23, 24]. Thus variation in the use of medicinal plants growth form might be associated with the difference in socio-cultural believes, ecological status and variations in the practices of traditional healers.

**Fig. 4:**
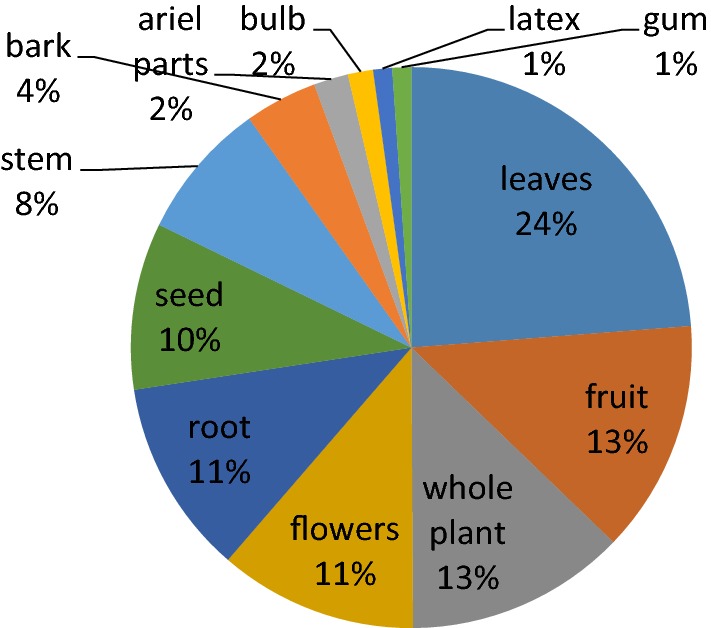
Plant parts used as medicine

It has also been revealed after literature review that leaves (131) are most commonly used in ethno medicine for the treatment of respiratory disorders. While other plant parts use frequencies are as follows, fruit (74), root (62), seeds (53), whole plant (70), stem (44), flowers (63), bark (23), and latex (6), and gum (6). Frequency of use of different plant parts are shown in Fig. 4. Preference of leaves over the other plant parts is commonly thought to be due to the reason that leaves are the photosynthetic organs containing the photosynthates which might be accountable for their medicinal values [25, 26]. It is may be due to the reason that the collection of leaves does not affect the life cycle of plant so it is preferred to use the leaves in ethno-preparations [27]. Fruit was the second most commonly used plant part according to the literature of Pakistan. It has also been reported in different studies that Fruit is being commonly used by Americans as well [28]. Roots were the third frequent used plant parts which may be due to the reason that active constituents are rich in roots [29, 30] but the collection of underground parts of the plant is not viable as it affects the plants life and such plants are considerd as highly threatened [31, 32]. The overview of ethno-botanical literature of whole country reveals that different plant parts are being used of the same plant in different areas which is may be due to the availability, ease of collection or ethnic believes of local people.

### Use and mode of preparation

**Fig. 5:**
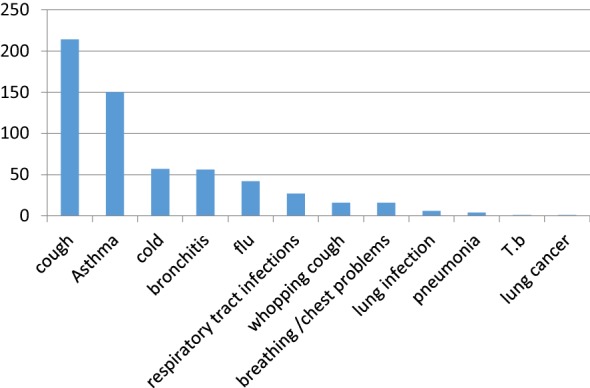
Common respiratory diseases treated traditionally

The reported plants were used in 12 different respiratory disorders. The highest number of medicinal plants documented are being employed in the treatment of cough (214) followed by asthma (150), cold (57), bronchitis (56), flu (42), respiratory tract infections (27), whopping cough (16) and breathing problems (16). Percentage of plants used in ethno-medicine for the treatment of different respiratory conditions is shown in Fig. 5.

**Fig. 6:**
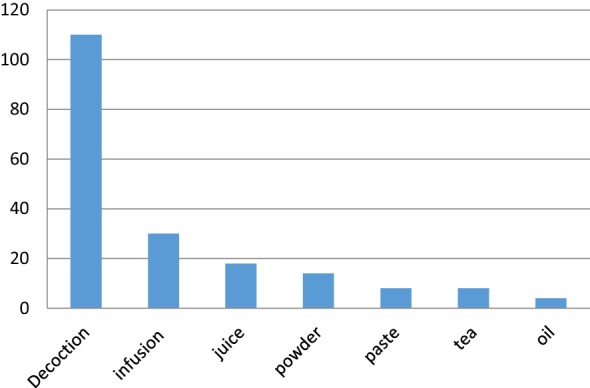
Mode of preparation of plant

Different recipes are being used by local peoples for the use of medicinal plants but most common mode of preparations of phytomedicines are decoction (110), powder (14), juice (18), infusion (30), paste (8), tea (8) and oil (4). Different mode of preparation of the plants is shown in Fig. 6. Decoctions are prepared by boiling the plant in water until the volume of water is reduced to half. Previously studies reported that decoction and infusion predominates [33] because these preparations are rapid to prepare, inexpensive and easy to consume. In addition, high usage of decoction might be related to their proven efficacy over many years’ trial and indigenous knowledge accumulated on effectiveness of such preparations.

Even though the literature was scrutinized exhaustively, there was missing information regarding the mode of preparation of ethno medicine. Many ethno-botanical studies published include raw lists of plants used medicinally, only indicating which parts are used for what ailments. Detailed preparation and application are rarely mentioned. These types of studies do not reveal the ideas underlying the use of the cited medicines nor do they explain why certain plants were selected. It is necessary to include precise medicinal indication for each species in future ethno-botanical studies in order to be able to reproduce the traditional preparations and understand their modes of actions. For example, individuals of the same species collected at different localities, in different seasons, even various times of the day and night or at different stages of growth might have strikingly different levels of active compounds [34]. Comprehensive information about both the collection and handling of each plant remedy needs to be meticulously recorded.

### Pharmacological evaluation and herbal preparations

An attempt has been made to investigate the pharmacological activity of the enlisted plants from available literature to confirm their traditional use against respiratory conditions. Only 53 plants out of 384 have been found on which pharmacological studies were conducted to evaluate their anti-inflammatory, immunomodulatory, smooth-muscle relaxants, anti-allergic, anti-histaminic, mast cell stabilizing, bronchodilator and antioxidant properties as these properties are useful for the treatment of respiratory conditions [35]. Remaining 331 species mentioned in the table still needs to be pharmacologically evaluated in order to confirm their folkloric claim. Medicinal plants evaluated for pharmacological effects have been mentioned in Table 3.

**Table 3:** Pharmacological evaluation of medicinal plants

Scientific name	Family	Part used	Traditional use	Pharmacological effect	Refs.
*Abies pindrow*	Amaranthaceae	Leaves	Cough, asthma and other chest infection	Brochoprotective against histamine-induced bronchospasm, cromoglycate-like action on the mast cells and inhibition of sensitization and synthesis of reaginic-type antibodies	[40]
*Abrus precatorius*	Leguminosae	Root and leaves	Asthma, cough	Anti-allergic and Mast cell stabilizing effect in egg albumin induced degranulation of mast cells	[137, 138]
*Achyranthes aspera* Linn.	Amaranthaceae	Leaves	Pneumonia and asthma	Antihistaminic, anti-inflammatory, mast cell stabilizer and bronchoprotective effect. Inhibits action of histamine, acetylcholine and 5-HT	[139, 140]
*Albizia lebbeck* Benth	Moraceae	Bark	Flu, cough, lung problems	Bronchodialator, Anti-allergic, Mast cell stabilizing property due to histamine release and cytokine expression of antigen –ige activated mast cells	[141, 142]
*Alstonia scholaris*	Fabaceae	Bark, Leaves	Asthma	Bronchodialator, anti-tussive effect against sulfur dioxide induced mice coughing and citric acid induced guinea pigs coughing. Anti-asthmatic activity against histamine induced bronchoconstriction	[143, 144]
*Althaea officinalis* L.	Lythraceae	Flowers, leaves, roots, fruits	Asthma and bronchitis	Cough suppressant activity in citric acid-induced cough reflex	[145]
*Artemisia vulgaris*	Asclepiadaceae	Leaves	Asthma	Bronchodilator due to anticholinergic and Ca^2+^ antagonist mechanisms	[146]
*Artemisia maritime*	Asteraceae	Aerial parts	Cough	Bronchodilator activities mediated possibly through dual blockade of calcium channels and phosphodiesterase	[102]
*Adhatoda vasica*	Capparidaceae	Whole plant	Cough, bronchitis, asthma	Antiallergic, anti-anaphylactic, anti-inflammatory, antitussive, bronchodilator and bronchoprotective activity	[147–149]
*Allium cepa*	Apiaceae	Stem, leaves	Cough	Mast cell membrane stabilizing activity through inhibitor of mast cell secretion and decrease in the release of tryptase, MCP-1 and IL-6	[150]
*Bauhinia variegata*	Mimosaceae	Bark, root, buds	Asthma	Mast cell membrane stabilizing effect	[151]
*Boerhavia procumbens*	Rutaceae	Roots	Cough, asthma	Anti-asthmatic and anti-inflammatory properties in Toluene diisocyanate (TDI) allergic model in rat	[152]
*Cynodon dactylon*	Ranunculaceae	Leaves	Asthma	Anti-anaphylactic activity and mast cell stabilizing activity	[153]
*Emblica officinalis*	Euphorbiaceae	Tree	Cold, cough	Anti-tussive activity	[37]
*Broussonetia papyrifera* (L.) Vent.	Moraceae	Fruit	Cough	Protective effect in lung inflammation and bronchitis	[154]
*Bunium persicum*	Apiaceae	Seeds	Cold, cough	Anti-histaminic effect	[155]
*Calotropis gigantean*	Convolvulaceae	All parts	Cough and asthma	Protective effect in anaphylaxis and allergic disorders due to antihistaminic and mast cell stabilizing effect	[156]
*Cannabis sativa*	Cannabiaceae	Whole plant	Cough	Bronchodilator	[157]
*Capparis spinosa* L.	Capparidaceae	Flowerl, seeds	Asthma, cough	Antiallergic and antihistaminic effect Bronchorelaxant effects in histamine-induced bronchospasm	[158]
*Capsicum annum* L.	Asteraceae	Fruit	Bronchitis	Bronchodilator	[159]
*Carum capticum* L.	Violaceae	Seeds, leaves and flowers	Cough bronchitis and diarrhea	Bronchodilatory and anti-cholinergic effect, anti-histamine effect	[160–162]
*Carum carvi* L.	Asteraceae	Stem and leaves	Bronchitis, cough	Bronchodilatory and anti-cholinergic effect	[163]
*Ephedra gerardiana*	Ephedraceae	Stem	Asthma/breathing problem	Anti-asthmatic activity in ovalbumin induced mice model	[164]
*Euphorbia hirta*	Labiatae	Whole plant	Asthma	Antihistaminic, antiallergic activity and mast cell from degranulation	[165]
*Ficus religiosa* L.	Papilionaceae	Fruit, leaves	Asthma	Mast cell stabilizing effect in histamine and acetylcholine induced bronchospasm model	[166]
*Inula racemosa*	Violaceae	Root	Asthma andbronchitis	Anti-histaminic, anti-serotonergic and Mast cell membrane stabilizing activity	[167]
*Lepidium sativum*	Fabaceae	Shoot	Cough and cold	Bronchodilator activity in histamine and acetylcholine induced bronchospasm model	[168]
*Mimosa pudica* Linn.	Lamiaceae	Roots, leaves	Asthma	Bronchodilator in histamine induced bronchospasm model	[169]
*Nyctanthes arbortristis*	Asclepidiaceae	Shrub flowers	Cough	Antihistaminic activity	[170]
*Glycyrrhiza glabra*	Adiantaceae	Roots	Cough	Demulscent, anti-tussive and anti-asthmatic effects via modulation of th1/th2 cytokines and enhancement of cd4+ cd25+ foxp3+ regulatory t cells in ovalbumin-sensitized mice	[35, 171]
*Helianthus annuus*	Papilionaceae	Flower, root, seed, leaves	Asthma, bronchial	Anti-asthmatic effect in Ovalbumin-induced mice	[172]
*Hyoscyamus niger* Linn.	Zygophyllaceae	Whole plant	Asthma, whooping cough	Bronchodilator effect through dual blockade of muscarinic receptors and Ca2+ channels	[173]
*Trachyspermum ammi*	Malvaceae	Seeds and oil	Bronchitis, asthma and colds, cough	Antiallergic, bronchodilator and spasmolytic effect (calcium antagonist)	[173]
*Mangifera indica*	Capparaceae	Flowers, leaves, bark and fruits	Asthma, cough	Anti-allergic, anti-asthmatic anti-cholinergic and anti-histamine effect	[174, 175]
*Murraya koenigii*	Rosaceae	Leaves	Asthma	Mast cell membrane stabilizing activity, anti-histaminic and anti-cholinergic effect	[3, 176]
*Nigella sativa*	Ranunculacea	Seeds	Whooping cough	Anti-histamine, bronchodilator and anti-asthmatic effect in asthmatic patients	[177–179]
*Ocimum basilicum*	Solanaceae	Leaves and seeds	Bronchitis, cough, cold	Bronchodilator and vasodilator activities through dual blockade of muscarinic receptors and Ca2+ channels	[180]
*Onosma bracteatum*	Moraceae	Whole plant	Asthma and bronchitis	Anti-allergic and anti-inflammatory action in bronchial hyperreactivity	[181, 182]
*Oryza sativa*	Salicaceae	Fruit, leaves, stem	Cold	Antianaphylactic effect, anti-inflammatory action by inhibition of histamine release from mast cells	[183, 184]
*Papaver nudicaule* L.	Papaveraceae	Herb	Cough	Cough and asthma-relieving effects in histamine phosphate induced asthma in guinea pigs	[185]
*Portula caoleracea*	Asteraceae	Aerial parts	Asthma	Bronchodilator, anti-tussive and anti-asthmatic effect in histamiin induced asthmatic model bronchodilator effect, anti-tussive and anti-asthmatic effect	[156, 186, 187]
*Ricinus communis*	Leporidae	Leaves	Asthma and cough	Antiasthmatic activity in milk induced leukocytosis and eosinophilic mice	[188]
*Salvia officinalis* Linn.	Liliaceae	Leaves	Cough, cold	Bronchodilator effect via activation of voltage-dependent K+ channels and inhibition of phosphodiesterase enzyme	[129]
*Solanum nigrum* L.	Solanaceae	Leaf, berries, flowers, root and stem	Cough, bronchitis	Mast cell stabilizing effect in milk-induced leucocytosis and eosinophilic mice	[189]
*Spinacia oleracea* L.	Amarylliadaceae	Leaves	Cough	Anti-asthmatic effect in ovalbumin-induced asthmatic model	[190]
*Taxus baccata* Linn.	Moraceae	Bark	Asthma and bronchitis	Protective effect against bronchoconstriction and bronchial hyperreactivity in e histamine and acetylcholine aerosol induced bronchospasm	[191]
*Viola odorata* L.	Berberidaceae	Whole Plant	Flu and cold	Anti-asthmatic effect and Bronchodilator	[192, 193]
*Artemisia scoparia* Waldst. and Kit.	Asteraceae	Whole plant	Cough, chest problems	Anti-asthmatic effect	[194]
*Vitexnegundo* Linn.	Verbenaceae	Leaves	Flu	Anti-asthmatic, anti-inflammatory, and anti-allergic mast cell stabilizing and bronchodilatory activity	[182]
*Cistanche tubulosa*	Poaceae	Whole plant	Cough	Mast cell membrane stabilizing activity, anti-allergic effect	[165, 190]
*Zingiber officinalis*	Asclepiadaceae	Stem, leaves	Flu	Anti-asthmatic anti-inflammatory and protection against LPS induced airway hyperreactivity	[195, 196]
*Ziziphus jujuba* Mill	Liliaceae	Fruits	Bronchitis	Anti-allergic and anti-anaphylactic activity, anti-histamine action in milk induced eosinophilia and leukocytosis	[197, 198]

Herbal formulations are the finished labeled products containing active ingredients or plant material or combination of medicinal plants [36]. With the increase in demand of traditional medicine, worth of herbal industry is also increasing day by day [37]. Local healers from different areas use different plants in various combinations to treat respiratory conditions. Some plants mentioned by tribal healers for the treatment of respiratory conditions are known to be used in the preparation of popular herbal medicines. Among such plants are *Achyranthes aspera, Adhatoda vasica, Glycyrrhiza glabra, Viola odorata* and *Onosma bracteatum*. The major domestic manufacturers like Hamdard, Qarshi, Ajmal and others produce 300–400 herbal products. A list of 17 commercially available herbal medicines used for respiratory conditions with their composition is mentioned in a Table 4. In recent time it is important to collect the valuable knowledge from local folklore regarding medicinal use of plants to treat respiratory conditions and give more focus on the useful pharmacological evaluation of medicinal plants for their protection, usefulness and effectiveness of this disease.

**Table 4:** Herbal products used for respiratory disorders

Sr. no.	Brand (manu-facturer)	Use /dose	Composition								
			*Ephedra gerardiana*	*Papaver somniferum*	*Achyranthes aspera*	*Glycyrrhiza glabra*	*Menthe arvensis*	*Valeriana officinalis*	*Mentha piperita*	*Hyssopus parvifloria*	*Zizphus vulgaris*
1.	Corezcol [1]	Expectorant/10 ml 6 times/day	✓	✓	✓	✓	✓				
2.	Hoopinil [1]	Cough/10 ml 6 times/day	✓		✓	✓			✓		
3.	Asthimna [1]	Asthma/10 ml TID	✓			✓					
4.	Expectum [1]	Expectorant/10 ml 6 times/day	✓			✓			✓	✓	✓
5.	Joshabasadar [2]	Cough/10 ml OD				✓	✓	✓			
6.	Linkus [3]	Cough/10 ml TID				✓		✓			
7.	Shaafijoshanda [3]	Cough/1 sachet flu/BID				✓		✓	✓		
8.	Sualin [2]	Cough, flu/1–2 tablets TID				✓					
9.	Suduri [2]	Bronchitis/10 ml 6 times/day				✓					
10.	Joharjoshanda [1]	Cough/1 sachet flu/BID				✓					
11.	Tiryaq e nazla [2]	Cough, flu/6 gm OD		✓		✓			✓		
12.	Infuza [2]	Asthma/10 ml OD				✓					
13.	Joshina [2]	Bronchitis/1 sachet BID				✓					
14.	Sharbatsadar [1]	Bronchitis/10 ml TID	✓			✓					
15.	Surfali [4]	Cough/10 ml TID				✓					
16.	Joshanda [2]	Cough and flu/1 sacet TID				✓					
17.	Sharbat e banafsha [1]	Cough/30 ml BID									

Sr. no.	Brand (manu-facturer)	Use /dose	Composition							
			*Mentha arvensis*	*Ocimum basilicum*	*Adhatoda vasica*	*Onosma bracteatum*	*Viola odorata*	*Acacia arabica*	*Zizphus sativa*	*Foeniculum vulgare*
1.	Corezcol [1]	Expectorant/10 ml 6 times/day	✓		✓					
2.	Hoopinil [1]	Cough/10 ml 6 times/day			✓					
3.	Asthimna [1]	Asthma/10 ml TID			✓					
4.	Expectum [1]	Expectorant/10 ml 6 times/day			✓					
5.	Joshabasadar [2]	Cough/10 ml OD	✓	✓	✓	✓				✓
6.	Linkus [3]	Cough/10 ml TID			✓		✓			
7.	Shaafijoshanda [3]	Cough/1 sachet flu/BID			✓		✓			✓
8.	Sualin [2]	Cough, flu/1–2 tablets TID		✓	✓					
9.	Suduri [2]	Bronchitis/10 ml 6 times/day		✓	✓					
10.	Joharjoshanda [1]	Cough/1 sachet flu/BID		✓			✓			
11.	Tiryaq e nazla [2]	Cough, flu/6 gm OD				✓		✓		
12.	Infuza [2]	Asthma/10 ml OD								
13.	Joshina [2]	Bronchitis/1 sachet BID					✓		✓	
14.	Sharbatsadar [1]	Bronchitis/10 ml TID			✓					
15.	Surfali [4]	Cough/10 ml TID						✓		
16.	Joshanda [2]	Cough and flu/1 sacet TID		✓		✓	✓		✓	
17.	Sharbat e banafsha [1]	Cough/30 ml BID					✓			

(1) Qarshi industries [pvt] Ltd, (2) Hamdard laboratories Waqf Pakistan, (3) Herbion Pakistan Pvt Ltd, (4) Ashraf labs

### Conservation status

During the investigation of conservation status of medicinal plants used against respiratory conditions in Pakistan 51 plant species were evaluated through IUCN Red list categories and criteria. Among these species 5 were endangered, 1 critically endangered, 11 vulnerable, 14 rare, 16 least concern, 3 infrequent and 1 near threatened. Conservation status of 51 medicinal plants is mentioned in Table 5. Non-scientific and indiscriminate collection of medicinal plants in various parts of the area has led to the severe pressure on the availability of medicinal plants. Using the part like roots, rhizomes, bulbs could also be a severe threat for reproducing medicinal plants of the area. Unplanned collection, loss of habitat, increased exploitation and unsustainable harvesting, intensive grazing, and land leveling for agriculture, deforestation and erosion attack of pathogens were the major threats to the medicinal plants. According to IUCN threatened plant data base, about 32,000 species of plants are threatened with extinction. This figure represent 13% of estimated 250,000 of plants It is stated that rate of plant extinction has reached to one specie per day as a result of mentioned threats and it is considered 1000–10,000 time faster than that would occur naturally. If the trend remains constant, 60,000 and 100,000 plant species may disappear in the near future [38, 39]. So in order to save these medicinal plants some important measures should be taken. Government should distribute saplings each year among the villagers to plant them. Media should be used to save nature and its importance. Establishment of nurseries and botanical garden as well as local community awareness and involvement to protect these national assets will be the best conservation measure.

**Table 5:** Conservation status of plants

*Scientific name*	Family	Medicinal use	Conservation status	Population trend	Refs.
*Abies pindrow* Royle	Amaranthaceae	Cough, asthma	Least concern	Stable	[199]
*Acacia modesta* Wall	Acanthaceae	Cough, asthma	Endangerd	Persistent	[38]
*Aconitum chasmanthum*	Ranunculaceae	Cough, and asthma	Critically endangered	Decreasing	[200]
*Aconitum violaceum*	Ranunculaceae	Asthma, cough	Vulnerable	Decreasing	[200]
*Alstonia scholaris*	Fabaceae	Asthma	Least concern	–	[201]
*Arisaema flavum*	Berberidaceae	Cough, cold	Rare	Increased	[38]
*Arisaema jacquemontii*	Asclepiadaceae	Asthma	Rare	Increased	[38]
*Artemisia scoparia*	Asteraceae	Cough chest problems	Rare	Increased	[38]
*Avena sativa*	Poaceae	Asthma	Infrequent	Decreasing	[202]
*Berberis lyceum*	Adiantaceae	Cough	Vulnerable	Increased	[82]
*Bergenia ciliate*	Apiaceae	Coughs and colds, asthma	Engangerd	Increased	[82]
*Bistorta amplexicaulis*	Polygonaceae	Flue.	Endangerd	Persistent	[82]
*Bunium persicum*	Apiaceae	Cold, cough	Rare	Increased	[82]
*Celtis australis* L.	Ulmaceae	Cough	Engangerd	Persistent	[38]
*Cichorium intybus* L.	Asteraceae	Asthma and breathing problems	Rare	Increased	[38]
*Cupressus sempervirens*	Asteraceae	Flu and cold	Least concern	Unknown	[203]
*Daucus carota*	Moraceae	Asthma, bronchitis	Infrequent	Decreasing	[204]
*Desmostachya bipinnata*	Nyctaginaceae	Asthma	Least concern	Unknown	[205]
*Ephedra gerardiana*	Ephedraceae	Asthma/breathing problem	Vulnerable	Increased	[82]
*Ephedra intermedia*	Rosaceae	Asthma and tuberculosis.	Least concern	Stable	[206]
*Ficus carica* L.	Moraceae	Cough	Least concern	–	[207]
*Ficus elastic*	Moraceae	Cough, asthma	Rare	Increased	[38]
*Ficus palmata*	Myrtaceae	Expectorant	Rare	Increase	[38]
*Inula grantioides*	Asteraceae	Asthma	Rare	Increased	[38]
*Juglans regia*	Euphorbiaceae	Asthma	Near threatened	Decreasing	[207]
*Juniperus excelsa*	Cupressaceae	Chest infection	Least concern	Stable	[203]
*Lemna minor*	Convolvulaceae	Cold	Least concern	Unknown	[208]
*Mangifera indica*	Capparaceae	Asthma, cough	Infrequent	–	[201]
*Mentha longifolia*	Labiateae	Cough	Rare	Increased	[82]
*Mimosa pudica* L.	Lamiaceae	Asthma	Least concern	Stable	[209]
*Morus alba* L.	Solanaceae	Cough	Vulnerable	Persistent	[38]
*Morus nigra* L.	Poaceae	Cough	Vulnerable	Persistent	[38]
*Olea ferruginea*	–	Cough, cold, flue	Endangerd	Persistent	[38]
*Opuntia dillenii*	Lythraceae	Asthma, whooping cough	Least concern	Stable	[210]
*Opuntia monacantha*	Caesalpinaceae	Bronchitis and asthma	Least concern	Stable	[211]
*Phyla nodiflora*	Verbenaceae	Cold	Least concern	Stable	[212]
*Pinus roxburghii*	Solanaceae	Coughs, cold	Least concern	Stable	[38]
*Pistacia integerrima*	Rosaceae	Cough, asthma	Vulnerable	Increased	[82]
*Plantago lanceolata*	Plantaginaceae	Cough and chest diseases	Rare	Increased	[82]
*Punica granatum*	Punicaceae	Cough	Least concern	–	[207]
*Pyrus communis* L.	Rosaceae	Cough	Vulnerable	Persistent	[38]
*Rhynchosia minima*	Mimosaceae	Asthma	Least concern	Stable	[207]
*Rubus fruiticosus*	Violaceae	Whooping cough	Vulnerable	Persistent	[38]
*Salvia nubicola*	Labiateae	Cough, asthma	Vulnerable	Persistent	[38]
*Thymus linearis*	Labiateae	Cough, asthma	Rare	Increased	[82]
*Thymus serpyllum* L.	Lamiaceae	Whooping cough, asthma	Vulnerable	Persistent	[38]
*Verbascum thapsus* L.	Scrophulariaceae	Cough	Rare	Persistent	[38]
*Viola serpens*	Lamiaceae	Lung trouble	Vulnerable	Persistent	[38]
*Viola biflora* L.	Pteridaceae	Cold and flu	Rare	Increased	[82]
*Viola canescens*	Violaceae	Cough	Rare	Increased	[82]
*Vitis vinifera* L.	Rhamnaceae	Bronchitis	Least concern	–	[213]

### Commercially available important plants

Among the 384 plants used against respiratory diseases 58 plants belonging to 32 families were commercially important and are a source of income for the local community. These plants are used as drugs for treating respiratory diseases in traditional system of medicine. The detailed list of local uses, part used price and commercial status for each plant is mentioned in the Table 6. The prices of each species vary from year to year and also depend on demand and supply. There was an increase of three to fivefolds in prices from collectors to the national market [40].

**Table 6:** Economical value of plants

Scientific name	Local name	Family	Part exported	Traditional use	Price/kg	Commercial status/exported to	Refs.
*Acacia nilotica*	Kikar	Apocynaceae	Flowers	Asthma	40	Increased	[214]
*Achyranthes aspera* Linn.	Puthkanda	Amaranthaceae	Rhizome/fruit	Pneumonia and asthma	–	Increased	[55]
*Aconitum chasmanthum*	Baroboma	Ranunculaceae	Roots	Cough, and asthma	25	Increased	[215]
*Aconitum heterophyllum*	Shaowboma	Ranunculaceae	Roots	Cough, asthma	400	Increased	[82, 215 ]
*Aconitum violaceum*	Bezhumolo	Ranunculaceae	Rhizome	Asthma, cough	250	Persistent	[55, 82]
*Adhatoda vasica*	Bansa	Capparidaceae	Leaves	Cough, bronchitis, asthma	5	Persistent	[215]
*Adiantum capillus-veneris*	Hansraal	Liliaceae	Whole plant	Coughs, bronchitis	250	Increased/Germany, Scotland, Iran and India	[82, 215 ]
*Adiantum incisum* Forssk	Pershoofa	Liliaceae	Fronds	Cough and cold	–	Increased/Germany, Scotland, Iran and India	[55]
*Adiantum venustum*	Sumbal	Liliaceae	Whole plant	Cough	5	Increased	[82]
*Allium sativum*	Thoom	Euphorbiaceae	Bulbs/leaves	Respiratory tract infection		Increased	[55]
*Ammi visnaga*	Chalveray	Amaranthaceae	Fruit	Bronchial asthma, breathing problems	40	Increased	[82]
*Artemisia vulgaris*	Baniru	Asclepiadaceae	Leaves/shoot, root	Asthma	12	Increased	[49, 55]
*Atropa acuminataroyle*	Lubbhar	Ranunculaceae	Whole plant	Cold, flu	12	Increased	[215]
*Berberis lyceum*	Kashmal	Papilionaceae	Wood roots	Cough	25	Increased	[82, 215 ]
*Bergenia ciliate*	Shaphus	Apiaceae	Leaves, rhizome, roots	Coughs and colds, asthma	300	Increased/China	[55]
*Bistorta amplexicaulis*	Saag	Polygonaceae	Rhizome	Flu	300	Persistent/Europe and India	[55, 82]
*Bunium persicum*	Zeera	Apiaceae	Fruit	Cold, cough	1000	Increased	[40, 55]
*Calotropis procera*	Akk	Asclepiadaceae	Roots, flowers	Cough	1000	Increased	[214]
*Cannabis sativa*	Bung	Cannabiaceae	Whole plant	Cough	10	Increased	[70]
*Capsicum annum*	Subzmirch	Asteraceae	Fruits	Bronchitis	–	Increased	[55]
*Carum carvi*	Sounjmik	Asteraceae	Seeds	Bronchitis, cough	160–180	Increased	[134]
*Carum copticum*	Ajwaindesi	Violaceae	Seed	Whooping, cough	15	Increased	[215]
*Cassia fistula*	Amaltas	Moraceae	Poods	Cough and flue	15	Increased	[215]
*Cicer arietinum*	Cholay	Myrtaceae	Fruit	Flu	40	Increased	[214]
*Cichorium intybus*	Qarali	Asteraceae	Leaves	Asthma and breathing problems	13	Increased	[49, 82]
*Citrullus colocynthis*	Bitter apple	Cucurbitaceae	Fruit	Bronchial asthma	50	Increased	[214, 215]
*Colchicum luteum*	Suranjaan-e-talkh	Colchicaceae	Coms	Bronchial diseases	500	Increased/Germany, South Africa, France and Bulgaria	[40, 215]
*Coriandrum sativum*	Dhaniya	Brassicaceae	Leaves/fruit	Respiratory tract infection	25	Increased	[55, 82]
*Diospyros lotus*	Amlok	Punicaceae	Fruits/leaves	Cough	300	Increased	[40]
*Ephedra gerardiana*	Soom	Ephedraceae	Fruit/leaves	Asthma/breathing problem	–	Increased	[55]
*Ephedra intermedia*	Somani	Ephedraceae	Bark/leaves	Asthma and tuberculosis	8	Increased	[82]
*Eucalyptus globulus*	Safaida	Moraceae	Bark/leaves	Flue	110	Increased	[214]
*Foeniculum vulgare*	Sonf	Papilionaceae	Leaves/seeds	Cough, pneumonia	20	Increased	[55, 214]
*Fumaria indica*	Pithpapadah	Fumariaceae	Shoot	Cough	–	Increased	[55]
*Hyoscyamus niger*	Khoobkalan	Zygophyllaceae	Seeds	Asthma, whooping cough	20	Increased	[215]
*Juglans regia*	Akhrot	Euphorbiaceae	Bark/fruit	Asthma	30	Increased	[55, 82]
*Mentha longifolia*	Jangli Podina	Labiateae	Whole plant	Cough	–	Increased	[55]
*Origanum vulgare*	Ganeyar	Violaceae	Root	Colds, flu, asthma	7	Increased	[82]
*Peganum harmala*	Harmal	Solanaceae	Seeds, leaves	Asthma	50	Increased	[214]
*Pistacia integerrima*	Kangar	Rosaceae	Leaves	Cough, asthma	–	Increased	[55]
*Plantago lanceolata*	Smanharswa	Plantaginaceae	Leaves/seeds	Cough and chest diseases	–	Increased	[55]
*Punica granatum*	Anar	Punicaceae	Fruit/bark/leaves	Cough	–	Increased	[55]
*Portulaca oleracea*	–	Asteraceae	Seeds/leaves	Asthma	–	Increased	[55]
*Rheum australe*	Chontal	Polygonaceae	Rhizome/leaves	Cough	10	Increased	[55, 82]
*Salvia moorcroftiana*	Gadakan	Malvaceae	Leaves/seeds	Cough	–	Increased	[55]
*Sisymbrium irio*	Khubkalan	Brassicaceae	Seeds	Cough	50	Increased	[214, 215]
*Skimmia laureola*	Nyra	Solanaceae	Leaves	Asthma	12	Increased	[82]
*Solanum surratense*	Kundiyara	Solanaceae	Fruits	Cough, asthma	–	Increased	[55]
*Tamarix dioica*	Rukh	Acanthaceae	Bark	Cough	100	Increased	[214]
*Thymus linearis*	Tumburu	Labiateae	Fruits	Cough, asthma	–	Increased	[55]
*Trachyspermum ammi*	Ajwain	Malvaceae	Fruits	Asthma and colds, cough	550	Increased	[40]
*Viola biflora*	Lilio	Pteridaceae	Flower	Cold and flu	16	Increased	[55, 82]
*Viola canescens*	Banafsha	Violaceae	Whole plant	Cough	16	Increased	[55]
*Viola serpens*	Banafsha	Violaceae	Flowers leave	Lung trouble	100–240	Increased/India, Germany and Scotland	[82, 215 ]
*Withania coagulans*	Akri	Meliaceae	Fruit	Cough, asthma	5	Increased	[215]
*Withania somnifera*	Asgand	Zingerberacee	Roots	Flu	35	Increased	[55, 215]
*Zizyphus nummularia*	–	Solanaceae	Fruit	Bronchitis	30	Increased	[214]
*Zizyphus sativa*	Mark Hany	Rhamnaceae	Fruit	Bronchitis	30	Increased	[55, 214]

In 2006 global trade of medicinal plants reached US$ 60 billion. Europe alone annually imports about US$ 1 billion from Africa and Asia. Such trade is expected to expand substantially by the year 2050 because of the increasing popularity of herbal medicines. Pakistan exports of high value plants generate over US$ 10.5 million annually in 2012, with a substantial percentage of the supply coming from Swat District [41]. Approximately 300 plant species are being traded in Pakistan of these were 22 medicinal plant species worth 14.733 million Rs were traded in 1990 while it was increased by 8.5 folds (122 million Rs). The geographical location of Swat District provides an ideal physical environment for the growth and nourishment of many high value medicinal and aromatic plants. These medicinal plants can make a contribution to the economic development of the area in particular and the country in general [41].

Market share of Pakistan has been declining due to unreliable and often poor quality of the material supplied, length of the supply chain, and poor marketing strategies. The availability of medicinal plants drastically decreased due to increased marketing pressure on medicinal plants, lack of job opportunities in the area, non-sustainable harvesting methods like digging of whole plant and increased population of the area. So to maximize the exports and benefits of medicinal plants, trade monitoring, equitable sharing of benefits of wild resources, improved control on harvesting and trade for the conservation of resources, enhancement of cultivation efforts, future research into trade in wild harvested plants, community participation in natural resource management and value addition in the herbal products are recommended [42].

## Future recommendations

In this review, we described the medicinal plants used in Pakistan to treat respiratory disorders. Local people are using plants without any scientific base. There is a gap between traditional use of plants and pharmacological evaluation as well as very limited number of phytochemical studies has been documented. In recent time it is important to collect the valuable knowledge from local folklore regarding medicinal use of plants to treat respiratory conditions and give more focus on the useful pharmacological and phytochemical evaluation of medicinal plants for the isolation of novel compounds as well as for their protection, usefulness and effectiveness of this disease. We examined investigated areas across Pakistan in relation to medicinal plants richness and based on this we provide recommendations for the areas that should be targeted in future ethno-botanical surveys. From the review of literature it is deduced that proper documentation of data was lacking in several research articles studied. There were many spelling mistakes in the plant names and families. Life form, part used and mode of preparation for herbal remedies were also not stated in many published ethno-medicinal surveys. So in future ethno-pharmacological research with comprehensive information should be carried out in the under investigated areas to save the traditional knowledge and to take it to the light of science. Appropriate measures should be taken to increase the market share of Pakistan as well as to maximize the exports and benefits of medicinal plants. Properly monitored trade and marketing for stability in product supply, unbiased sharing of profits of wild resources, improved control on harvesting and trade for the conservation of resources, enhancement of cultivation efforts, creation of new markets for various products so as to profit the public, implementing rules and regulations at public level to facilitate attaining goals of economic development and ecosystem conservation, community participation in natural resource management and value addition in the herbal products are recommended.

The problems of biodiversity loss can be solved by underlying recommendations

- Government should distribute saplings each year among the villagers to plant them.
- Media should be used to save nature and its importance.
- Initiating afforestation projects and controlling over grazing.
- Reducing biotic pressure by supplying gas and electricity.
- Establishment of nurseries and botanical garden as well as local community awareness and involvement to protect these national assets will be the best conservation measure.
- Commercial exploitation of medicinal plants should make sure to safeguard the intellectual property rights of local people.
- Providing educational material in native languages to update collectors about occurrence of medicinal flora, their therapeutic significance, and market values.
- It is important to identify valuable species, precisely map their distribution, document their status, study their life cycle, and formulates guidelines for their conservation and management.

## Conclusions

In essence, the current investigation identified that people from Pakistan discern and make use of 384 therapeutic plants, belonging to 85 families for respiratory disorders. Keeping in view the results, Asteraceae family contains more plants and herbs are the dominant life form, whereas among the parts, leaves have been maximally used in decoction form for the treatment of respiratory disorders. Moreover, 17 plant species are being frequently used by the manufacturers in different herbal products for the treatment of respiratory disorders and only 53 plants have been pharmacologically evaluated while 51 plants are in the IUCN threatened list as well as 58 plant species have reasonable commercial significance. This review will not only provide a baseline data for initial screening of promising plants used in respiratory disorders but also will be helpful for conducting phytochemical studies by the application of ethno botanical indices. The study also provides recommendations for the areas that should be targeted in future ethno-botanical surveys. The need of hour is to implement productive policies for the careful use of valuable ethno botanical inheritance of Pakistan and to fill the gap between ethno-medicine and pharmacological research, to fully elucidate promising significances of plant-derived medicines on public health.
